# SARS-CoV-2 Antibody Dynamics in Healthcare Workers after mRNA Vaccination

**DOI:** 10.3390/vaccines11020358

**Published:** 2023-02-04

**Authors:** Kevin D. Dieckhaus, Min-Jung Kim, Jian-Bing Shen, Tina S. Liang, Michael J. Kleinberg, Kristen M. Siedlarz, David B. Banach, Mark L. Metersky, Rob P. Fuller, Eric M. Mortensen, Bruce T. Liang

**Affiliations:** UConn Health, Farmington, CT 06030, USA

**Keywords:** SARS-CoV-2, COVID-19, neutralizing antibody, bnt162b2, mRNA-1273, healthcare workers

## Abstract

Since the emergence of SARS-CoV-2, maintaining healthcare worker (HCW) health and safety has been fundamental to responding to the global pandemic. Vaccination with mRNA-base vaccines targeting SARS-CoV-2 spike protein has emerged as a key strategy in reducing HCW susceptibility to SARS-CoV-2, however, neutralizing antibody responses subside with time and may be influenced by many variables. We sought to understand the dynamics between vaccine products, prior clinical illness from SARS-CoV-2, and incidence of vaccine-associated adverse reactions on antibody decay over time in HCWs at a university medical center. A cohort of 296 HCWs received standard two-dose vaccination with either bnt162b2 (Pfizer/BioNTech) or mRNA-1273 (Moderna) and were evaluated after two, six, and nine months. Subjects were grouped by antibody decay curve into steep antibody decliners gentle decliners. Vaccination with mRNA-1273 led to more sustained antibody responses compared to bnt162b2. Subjects experiencing vaccine-associated symptoms were more likely to experience a more prolonged neutralizing antibody response. Subjects with clinical SARS-CoV-2 infection prior to vaccination were more likely to experience vaccination-associated symptoms after first vaccination and were more likely to have a more blunted antibody decay. Understanding factors associated with vaccine efficacy may assist clinicians in determining appropriate vaccine strategies in HCWs.

## 1. Introduction

The SARS-CoV-2/COVID-19 pandemic has led to significant morbidity and mortality, with greater than 550 million infections and six million deaths [1]. The impact of the COVID-19 illness on healthcare workers (HCWs) is profound. As of May 2021, the World Health Organization estimated over 80,000 COVID-related deaths amongst HCWs worldwide [2]. The incidence of COVID-19 in HCWs may mirror the prevalent community environment and local epidemiology [3,4,5,6,7,8,9]. Additional occupational exposure of HCWs has been postulated to be a significant risk factor for acquisition of SARS-CoV-2 in some settings [10,11,12,13,14]. Maintaining protection of HCWs through appropriate infection control methods and vaccination remains critical to maintaining a public health and clinical care workforce in order to manage the pandemic [15]. In December 2020, vaccination programs prioritizing healthcare workers for vaccination with the bnt162b2 vaccine (Pfizer/BioNTech) and the mRNA-1273 vaccine (Moderna) were initiated in the United States. Vaccination leads to development of antibody targeting the SARS-CoV-2 spike protein, the primary target of neutralizing activity during viral infection, and it confers protection against symptomatic infection, hospitalization, and death. Antibody responses to both vaccination and clinical illness diminish over time, which may make hosts at increased risk for subsequent infection. Neutralizing antibody activity after vaccination may be retained despite quantitative decreases in total spike protein antibody [16]. Both natural infection [17] and mRNA vaccination [18] elicit antibody production to the receptor binding domain within the S protein, leading to inhibition of viral entry into host cells, conferring reduced rates of symptomatic COVID-19 in health care personnel [19]. Reinfections may be more prevalent in healthcare worker populations [20] compared to community cohorts. Thus, understanding vaccine response rates and correlates of immune response remain important for understanding the ongoing impact of COVID-19 on the HCW workforce.

## 2. Materials and Methods

### 2.1. Study Design, Recruitment, and Participants

A total of 296 healthcare workers employed at UConn Health, Farmington, Connecticut, were enrolled in this longitudinal and ongoing study, beginning in February 2021. The study was approved by the University of Connecticut Institutional Review Board (#20-186-1). Informed consent was obtained from all subjects involved in the study. The study was performed in accordance with Helsinki Declaration and followed Good Laboratory Practice. We collected blood samples and conducted clinical interviews at months two, six, and nine after HCWs received a two-dose primary vaccination with either bnt162b2 or mRNA-1273 mRNA vaccines, with an additional interview conducted at month twelve. Vaccine availability at time of initial vaccination was limited. When more than one vaccine product was available, the product given was based on vaccine availability and HCW preference. Study timing correlated to evaluating vaccine response from an initial two-dose vaccination strategy, prior to 3rd dose vaccine boosting, which became available to HCWs at approximately month 10 of analysis. The timing of this evaluation correlated with the community transmission of the SARS-CoV-2 ancestral, alpha, and delta variants.

### 2.2. Laboratory Methods

Semi-quantitative analysis of SARS-CoV2 cPass Surrogate Virus Neutralizing Test kit (GenScript, Piscataway, NJ, USA, #L00847) was performed according to the manufacturer’s instructions. The kit utilized the recombinant receptor binding domain (RBD) of the SARS-CoV-2 spike protein to detect antibodies that specifically block the RBD from binding to the hACE2 receptor. Samples and standards were tested in duplicate. A neutralizing antibody standard curve was designed to validate the kit and to semi-quantitatively assess neutralizing antibody titers in diluted samples. Monoclonal neutralizing antibodies (IgG/IgM positive control mixture) were diluted in negative control matrix to a stock concentration of 10 μg/mL, followed by serially diluted 1:2 in negative matrix control. Since each dilution was mixed with an equivalent volume of RBD conjugated horseradish peroxidase (RBD-HRP), the final, starting concentration for the standard curve was 5 µg/mL. Negative matrix control alone was used for background wells, and the kit supplied a negative control. Serum samples, as well as positive and negative controls, were diluted at a ratio of 1:10 with sample dilution buffer. The same amount of standards, samples, and controls were mixed with horseradish peroxidase conjugated recombinant SARS-CoV-2 RBD solution and incubated for 30 min at 37 °C. The mixtures were subsequently incubated for 15 min at 37 °C in a capture plate that was pre-coated with hACE2 protein. After a washing step, tetramethylbenzidine (TMB) solution was added, and the plate was incubated in the dark at room temperature for 15–18 min. Stop solution was added to quench the reaction, and the absorbance was immediately read at 450 nm on an ELISA microplate reader (Agilent Technologies, Santa Clara, CA, USA). For semi-quantitative analysis, all the 450 nm readings from the tested serum samples were then plotted and interpolated against the standard curve to determine a titer value that, after accounting for the dilution factor, gave a final neutralizing titer per sample.

### 2.3. Clinical Assessment

Vaccine-related adverse events (AEs) were assessed and graded according to four severity levels (None/Mild/Moderate/Severe) based on the Common Terminology Criteria for Adverse Events (CTCAE) [21]. Baseline COVID-19 status was assessed by history of a documented positive SARS-CoV-2 PCR. Breakthrough infection was defined as a symptomatic clinical course consistent with COVID-19, with a documented positive SARS-CoV-2 PCR, ninety days or later from time of second vaccination of the primary vaccine series.

### 2.4. Aims

The primary aim was to evaluate the immunogenicity of the vaccines and decay of neutralizing antibody titers in vaccinated healthcare workers who may have experienced direct or indirect care-related exposure to suspected or known COVID-19 patients in addition to ongoing community exposures. Secondary aims were to assess correlation of infection rates and re-infection in the vaccinated cohort with vaccine response, the impact of clinical SARS-CoV-2 illness on antibody dynamics, and the relationship between vaccine-associated symptoms and antibody response dynamics.

### 2.5. Statistical Methods

All baseline characteristics, antibody levels, and vaccine-associated symptom survey data were visually inspected and assessed for normality using the Anderson-Darling test to determine appropriate statistical approaches. Variables were presented as means with standard deviations, and differences in variables by group were evaluated using two sample t-tests if the data satisfied normality assumption. Otherwise, descriptive statistics for variables were presented as medians with interquartile range (IQR) or count with percentage.

Baseline was defined as the date of 2nd vaccination with either bnt162b2 or mRNA-1273. The impact of individual vaccine and pre-vaccination COVID-19 infection status on antibody levels was evaluated in subjects, exclusive of values obtained after any breakthrough from vaccination through September 2021, as booster vaccinations became available for the HCW cohort in October 2021. The comparisons of antibody levels over time (Month 2, Month 6, and Month 9 after 2nd vaccination) were accomplished with the use of Kruskal-Wallis test with subsequent Dunn’s multiple comparisons. Wilcoxon Rank-Sum tests were employed to make a comparison of antibody levels and slopes from Month 2 to Month 9 by both baseline COVID-19 infection status and vaccine product.

Lastly, this study analyzed how AE profiles observed after 1st and 2nd vaccination correlated with different clinical histories of COVID-19 infection prior to vaccination, specific vaccine products, and slope of antibody level decline. Subjects were classified into two groups each by (1) COVID-19 infection history at baseline (defined as prior COVID-19 illness or no history of infection), (2) vaccine product and (3) slopes of antibody levels from Month 2 to Month 9 (defined as a gentle decline or steep decline as compared to the median decline in antibody levels). Fisher’s exact tests and odds ratio with 95% confidence intervals (CI) were used to determine the associations between the presence of AEs and these three group factors. The data were statistically analyzed using SAS.9.2 and MATLAB R2021b, with a two-tailed significance level defined as *p* < 0.05.

## 3. Results

As of 11 May 2022, 296 participants completed this study with an average (STD) age of 43.7 (12.2) years; 248 (83%) were Caucasian, and 237 (80%) were female. There were no statistically significant differences in underlying health conditions between participants with or without a history of clinical COVID-19 at baseline. Eight subjects were moderately or severely immunocompromised [22]. Of the study participants, 159 (54%) received the bnt162b2 vaccine, and 137 (46%) received the mRNA-1273 vaccine. At baseline, 46 subjects (15.5%) reported prior PCR-confirmed clinical illness prior to vaccination. Of these subjects reporting prior illness products, 17 subjects received bnt162b2 and 29 subjects received mRNA-1273 (10.7% and 21.2% of bnt162b2 and mRNA-1273 recipients, respectively, *p* = 0.02) (Table 1).

### 3.1. Antibody Dynamics

**Figure 1:**
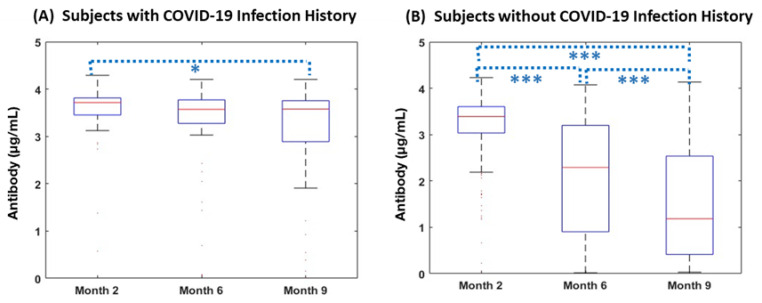
Changes in Antibody over Time per baseline COVID infection history. The following figures indicate (**A**) antibody responses (µg/mL) in individuals with COVID history before vaccination at Month 2 (n = 45), Month 6 (n = 44), and Month 9 (n = 34); and (**B**) antibody responses (µg/mL) in those without COVID history before vaccination at Month 2 (n = 224), Month6 (n = 227), and Month 9 (n = 143) after the 2nd vaccination. Levels of antibodies in both cohorts significantly decreased over time. Each box and whisker plot indicates the median, IQR, and range of antibody levels. *** *p* < 0.001, * *p* < 0.05.

We sought to evaluate changes in antibody level after vaccination. All groups demonstrated decreasing antibodies over time. However, those with no prior history of COVID-19 infection had a more precipitous decline in antibody levels over time compared to those with a prior history of COVID-19 infection prior to vaccination. Of 293 subjects without breakthrough before Month 9, 248 subjects (84%) were not previously known to be infected with SARS-CoV-2 at study entry. Neutralizing antibody was detectable in all COVID-naïve subjects at Month 2 but declined significantly by Month 6 and Month 9 with median (IQR) antibody titers of 3.39 (3.03–3.6), 2.29 (0.9–3.2), and 1.18 (0.41–2.54) µg/mL respectively (*p* < 0.001). A significant decrease in antibody levels was also noted from Month 2 to Month 6 (*p* < 0.001), Month 6 to Month 9 (*p* < 0.001), and Month 2 to Month 9 (*p* < 0.001). Subjects who had a history of prior COVID-19 infection also demonstrated a pattern of decreasing antibodies over the follow up period. The median (IQR) antibody level across Month 2, Month 6, and Month 9 were 3.72 (3.45–3.81), 3.57 (3.27–3.77), and 3.58 (2.89–3.76), respectively, with median antibody levels decreasing significantly from Month 2 to Month 9 (*p* = 0.04) (Figure 1). There were no differences in antibody levels between males and females at months 2, 6, or 9.

**Figure 2:**
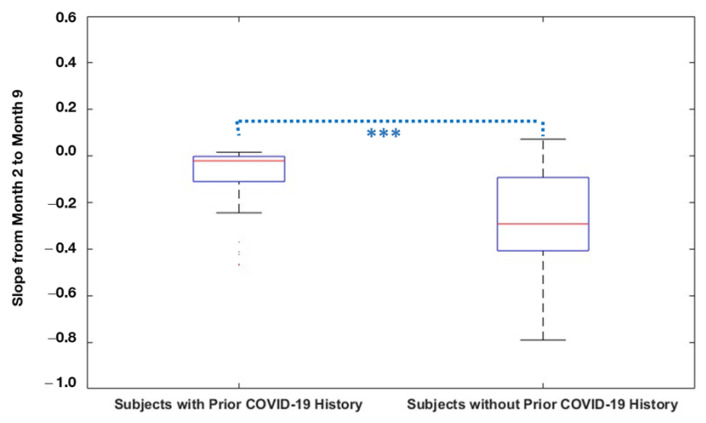
Antibody Decay Slope per baseline COVID infection history. Slopes in antibody levels from Month 2 through Month 9 were compared between subjects with COVID-19 history before vaccination (n = 46), and without COVID-19 history prior to vaccination (n = 249). The slope of antibody decline over time was significantly steeper in the subjects without a history of pre-vaccination COVID-19 infection. *** *p* < 0.001.

Comparing neutralizing antibody levels in subjects with and without prior infection with SARS-CoV-2, the subjects reporting prior infection had higher antibody levels compared to previously uninfected subjects at all time points. Antibody levels for subjects with prior COVID-19 infection history were significantly higher at Month 2 (*p* < 0.001), Month 6 (*p* < 0.001), and Month 9 (*p* < 0.001) compared to those without prior infection. The slope of antibody decline over time was significantly steeper in the subjects without a history of pre-vaccination COVID-19 infection (Median [IQR]: −0.29 [−0.41–−0.09]) compared to those with a COVID-19 infection history (Median [IQR]: −0.02 [−0.11–0]; *p* < 0.001). Each subject’s antibody decay curve was compared to the median slopes of decay, allowing classification as either a gentle decline or a steep decline; 85% of subjects reporting a history of COVID-19 infection displayed a gentle decline, whereas only 44% of those reporting no prior history of COVID-19 infection displayed a gentle decline in antibody levels over time (*p* < 0.001) (Figure 2, Table 2). There were no differences in antibody decay slope group between males and females.

### 3.2. Subjects without Pre-Vaccination COVID-19 Infection History

**Figure 3:**
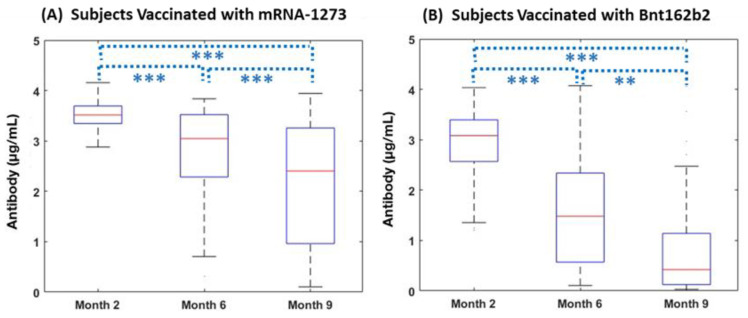
Changes in Antibody over Time by vaccine manufacturer for subjects without pre-vaccination COVID-19 infection history. (**A**) antibody responses (µg/mL) in individuals vaccinated with mRNA-1273 at Month 2 (n = 95), Month 6 (n = 91), and Month 9 (n = 69); and (**B**) antibody responses (µg/mL) in those vaccinated with bnt162b2 at Month 2 (n = 126), Month 6 (n = 133), and Month 9 (n = 71), amongst subjects without COVID infection history at baseline. Levels of antibody in both cohorts significantly decreased over time. *** *p* < 0.001, ** *p* < 0.01.

Out of 247 subjects without COVID-19 infection history at baseline and without breakthrough during this study, all showed a pattern of decreasing antibody over time (*p* < 0.001). Subjects vaccinated with bnt162b2 had medians (IQRs) of antibody levels of 3.29 (2.73–3.58), 1.7 (0.6–2.68), and 0.65 (0.14–1.31) at Month 2, Month 6, and Month 9, respectively. Subjects receiving mRNA-1273 also demonstrated a decreasing pattern over time (median [IQR]: 3.47 [3.3–3.65] at Month 2; 3.12 [2.12–3.5] at Month 6; and 2.07 [1.0–3.19] at Month 9; *p* < 0.001). Comparing COVID-19--naïve subjects vaccinated with bnt162b2 and mRNA-1273, subjects who received mRNA-1273 retained significantly higher neutralizing antibody levels over time. Antibody levels for subjects with mRNA-1273 were significantly higher at Month 2 (*p* < 0.001), Month 6 (*p* < 0.001), and Month 9 (*p* < 0.001) compared to subjects with Bnt162b2. At Month 9, neutralizing antibody levels were at 20% (Bnt162b2) and 69% (mRNA-1273) compared to values two months after the 2nd vaccination (*p* < 0.001) (Figure 3).

The slope of decline over time was significantly steeper in the Bnt162b2 vaccinated subjects (Median [IQR]: −0.35 [−0.42–−0.25] compared to those with mRNA-1273 (Median [IQR]: −0.13 [−0.3–−0.03] (*p* < 0.001). When subjects were categorized as either a gentle decline or steep decline compared to the median slope of decay, mRNA-1273 and bnt162b2 accounted for a “Gentle Decline” at 60% and 31%, respectively (*p* < 0.001) (Table 3).

### 3.3. Subjects with COVID-19 Infection History

**Figure 4:**
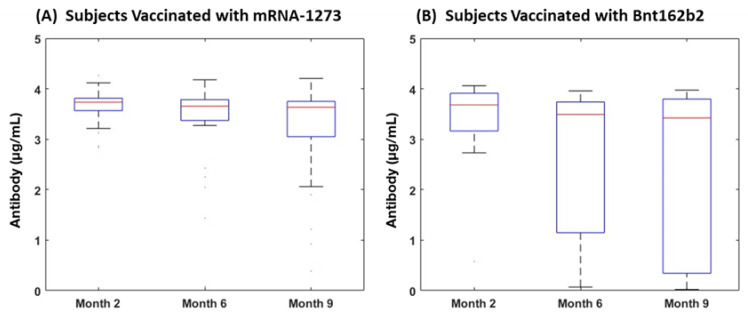
Changes in Antibody over Time per Vaccine Use for Subjects with COVID-19 Infection History. (**A**) Antibody responses (µg/mL) in individuals vaccinated with mRNA-1273 at Month 2 (n = 29), Month 6 (n = 29), and Month 9 (n = 25); (**B**) antibody responses (µg/mL) in those vaccinated with Bnt162b2 at Month2 (n = 16), Month 6 (n = 15), and Month 9 (n = 9) in subjects with a history of prior COVID-19 infection history at baseline. Levels of antibodies in both cohorts did not show statistical declines over time.

Of the 46 subjects reporting a clinical COVID-19 infection history at baseline who did not experience breakthrough during this study, those vaccinated with mRNA-1273 had a median (IQR) of antibody level of 3.73 (3.46–3.81), 3.65 (3.21–3.79), and 3.63 (3.05–3.75) at Month 2, Month 6, and Month 9, respectively. This group showed a pattern of decreasing antibodies over time, but changes were not statistically significant. Subjects receiving bnt162b2 also demonstrated a decreasing pattern, but had no significant differences over time (median [IQR]: 3.69 [3.45–3.8] at Month2; 3.49 [3.35–3.69] at Month 6; and 3.44 [0.44–3.78] at Month 9). Comparing subjects vaccinated with Bnt162b2 and mRNA-1273, antibody levels for subjects with mRNA-1273 were not statistically different those with bnt162b2 at Month 2, Month 6, and Month 9. The slope of the antibody decay curve from Month 2 to Month 9 also had no statistical differences by vaccine product. In this group, subjects vaccinated with mRNA-1273 and Bnt162b2 had a similar distribution into gentle and steep slope decline categories, with 85% characterized as having a gentle decline, including 83% of mRNA-1273 participants and 88% of bnt162b2 participants (*p* = 0.6) (Table 4, Figure 4).

### 3.4. Clinical Infections after Vaccination

A total of eight of 296 subjects (2.7%) developed clinical breakthrough infection with SARS-CoV-2 PCR positivity over the 12-month observation period, with five noted amongst previously naïve vaccine recipients (infection) and three amongst subjects previously infected with SARS-CoV-2 (reinfection). Amongst COVID-naïve vaccine recipients, the overall rate of breakthrough infections by month 12 was three of 108 mRNA-1273 recipients (2.8%) and two of 143 bnt162b2 recipients (1.4%). Three additional subjects who reported having prior COVID-19 infection at baseline developed reinfection after receiving mRNA-1273.

**Figure 5:**
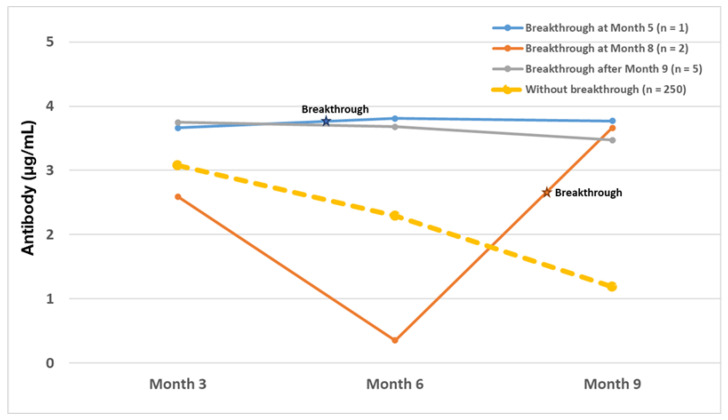
Antibody Titers in Breakthrough Subjects who had Subsequent Antibody Titer Determinations. Median antibody levels (µg/mL) for subjects without breakthrough infection after the 2nd vaccination (n = 250), subjects with breakthrough infection after Month 9 (n = 5), subjects with breakthrough infection at Month 8 (n = 2), and subjects with breakthrough infection at Month 5.

Of the eight breakthrough infections identified, there were three subjects who had breakthrough infections before the Month 9 visit. One (vaccinated with mRNA-1273) occurred at Month 5 (M5), with corresponding AB levels of 3.66, 3.81, and 3.77 for Month 2, Month 6, and Month 9 respectively. The other two cases (vaccinated with bnt162b2) occurred at Month 8. These both demonstrated a sharp increase in antibodies at Month 9, more than seven times the antibody levels at Month 6 (Figure 5). In the remaining five cases, the event occurred after antibodies were collected at Month 9; therefore, changes in antibody after infection could not be observed. Three of the breakthrough cohort were amongst the patients known to have a history of clinical COVID-19 prior to study initiation (incidence 6.52%), whereas subjects without a history of clinical COVID-19 infection at baseline had five clinical breakthrough infections (incidence 2.0%). Prior infection with SARS-CoV-2 at baseline was associated with an odds ratio (OR) of 3.42 (95% CI: 0.79–14.83) of breakthrough over the course of the observation period compared to those immunologically naïve at baseline, but this was statistically non-significant (*p* = 0.11).

### 3.5. Vaccine-Associated Adverse Events

We observed vaccine-related adverse events (AEs) after the 1st and 2nd vaccines. In general, vaccine-associated adverse events were mild and self-limited. In total, 1509 adverse events (AEs) were reported among 288 subjects (97% of the cohort). The overall AEs were classified as Grade I/mild (50%), Grade II/moderate (39%), and Grade III/severe (11%). Evaluating the patterns of AEs by each vaccination, AEs after the 2nd vaccination were more likely than after first vaccination, with 494 events reported after the 1st vaccination and 1015 AEs were reported after the 2nd vaccination (*p* < 0.001). In total, 87% reported at least one adverse effect after the 1st vaccination; 92% reported at least one adverse effect after the 2nd vaccination. After the 1st vaccination, the majority of AEs 57% were graded as mild, 37 % were graded as moderate, and 6% were graded as severe. After the second vaccination, 47% of AEs were graded as mild, 40% were graded as moderate, and 13% were graded as severe severity (*p* = 0.001). Women were more likely to experience an adverse event after the 1st vaccination (16% vs. 12%, *p* < 0.01) and 2nd vaccination (33% vs. 25%, *p* < 0.001). Women were also more likely to experience a grade 2 or higher adverse event than men after both the first vaccination (7% vs. 3%, *p* < 0.0001) and the second vaccination (18% vs. 12%, *p* < 0.0001).

The most frequent adverse event noted after vaccination was pain at the injection site, representing 86% of AEs reported after first vaccination and 79% after second vaccination. The incidence of taking antipyretic or anti-inflammatory medication was 24% after 1st vaccination and increased to 63% after 2nd vaccination (*p* < 0.001). Women were more likely to report medication use for symptoms (*p* = 0.03) after the 1st vaccination, as well as fatigue (*p* = 0.01) and headache (*p* = 0.03) after the 2nd vaccination.

Evaluating the impact of specific vaccine product on AEs, subjects vaccinated with mRNA-1273 were significantly more likely to develop a grade 1 or greater AE after the 1st vaccination (OR [95% CI]: 2.15 [1.01–4.55]; *p* = 0.04) and after the 2nd vaccination (OR [95% CI]: 3.01 [1.08–8.47]; *p* = 0.03) compared to subjects vaccinated with bnt162b2. Amongst subjects who were COVID-19-negative at baseline, those vaccinated with mRNA-1273 had a higher OR [95% CI] of fever (2.49 [1.35–4.57]; *p* < 0.001), fatigue (2.24 [1.29–3.9]; *p* = −0.005), headache (1.9 [1.14–3.15]; *p* = 0.02), chills (2.58 [1.52–4.31]; *p* < 0.001), muscle pain (2.16 [1.3–3.6]; *p* = 0.003), joint pain (1.96 [1.09–3.54]; *p* = 0.03), and use of antipyretic or anti-inflammatory medication (1.93 [1.13–3.3]; *p* = 0.02) after the second vaccination compared to subjects vaccinated with bnt162b2. There was no significant association between vaccine product and incidence or severity of AEs with first vaccination.

#### 3.5.1. Relationship of Vaccine-Associated Adverse Events and Antibody Decay Characteristics

**Figure 6:**
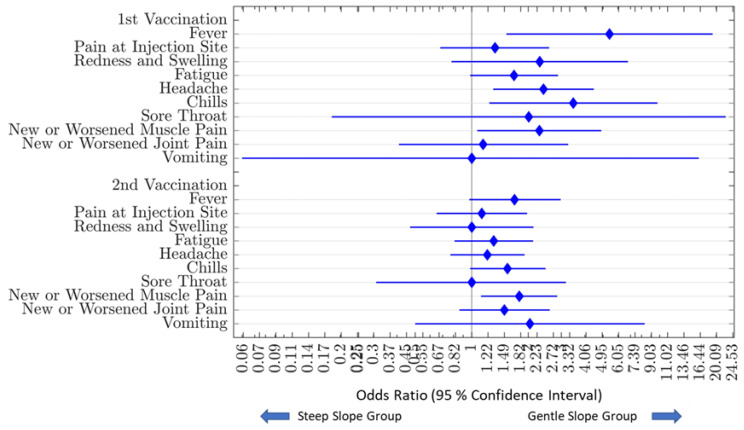
Association of Vaccine-Associated Adverse Events on Antibody Decay. Amongst all study subjects, the incidence of vaccine-associated adverse events between the gentle slope group (n = 148) and the steep slope group (n = 148) were compared. Those in the gentle slope group were significantly more likely to have fever (OR = 5.41), headache (OR = 2.41), chills (OR = 3.47), and new or worsening muscle aches (OR = 2.29) after the 1st vaccination and have new or worsening muscle aches (OR = 1.79) after the second vaccination. Blue diamonds represent the odds ratio of having reporting vaccine-associated adverse events. Bars to the left and right of each blue diamond represent the upper and lower confidence intervals.

Subjects were categorized based on antibody decay slope as either a gentle or steep decline compared to the median decay slope of the entire cohort over the observation period. Comparing the incidence of AEs between subjects in the two antibody decay slope groups, presence of fever (OR [95% CI]: 5.41 [1.53–19.12]; *p* = 0.01), headache (2.41 [1.3–4.46]; *p* = 0.01), chills (3.47 [1.24–9.73]; *p* = 0.02), and new or worsening muscle aches (2.29 [1.07–4.89]; *p* = 0.04) after the 1st vaccination, and new or worsening muscle aches (1.79 [1.12–2.85]; *p* = 0.02) after the 2nd vaccination, predicted a more sustained response with a more gentle decline in antibody levels over time. Other factors, including pain or erythema at the injection site, fatigue, headache, sore throat, new or worsening joint pain, vomiting, or diarrhea, were not associated with any statistical difference in antibody dynamics over time (Figure 6).

#### 3.5.2. Impact of Prior COVID Illness on Adverse Events and Antibody Dynamics

We observed that, after the 1st vaccination, subjects who reported prior COVID-19 infection at baseline (n = 46) had a higher OR [95% CI] of having fever (14.29 [CI: 5.05–40.59]; *p* < 0.001), redness and swelling (4.81 [CI: 1.69–13.65]; *p* = 0.005), fatigue (3.67 [1.9–7.07]; *p* = 0.001), headache (2.23 [CI: 1.09–4.54]; *p* = 0.03), chills (7.54 [CI: 2.99–19.06]; *p* < 0.001), new or worsening muscle pain (5.03 [2.31–10.94]; *p* = 0.001), new or worsening joint pain (4.02 [CI: 1.36–11.9]; *p* = 0.02), and taking antipyretic or anti-inflammatory medication (2.08 [1.06–4.06]; *p* = 0.04) compared to those who reported no such prior exposure. There were no significant differences in vaccine-associated adverse event profiles after the 2nd vaccination.

**Figure 7:**
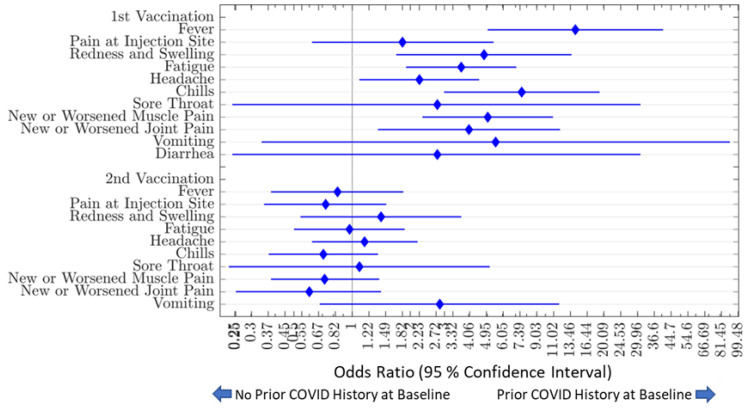
Odds Ratios of Vaccine-Associated Adverse Events Comparing Those With and Without Prior COVID Illness. Evaluating the associations between COVID infection history prior to vaccination and vaccine-associated adverse events, subjects who reported prior COVID infection at baseline (n = 46) were significantly more likely to have fever (OR = 14.29), redness and swelling (OR = 4.81), fatigue (OR = 3.67), headache (OR = 2.23), chills (OR = 7.54), new or worsening muscle pain (OR = 5.03), new or worsening joint pain (OR = 4.02), and taking antipyretic or anti-inflammatory medication (OR = 2.08) compared to those who reported no COVID-19 infection (n = 250) prior to vaccination. Blue diamonds represent the odds ratio of having reporting vaccine-associated adverse events. Bars to the left and right of each blue diamond represent the upper and lower confidence intervals.

In subjects who reported a history of COVID-19 infection prior to enrollment, development of fatigue (35%; *p* = 0.04) and chills (46%; *p* = 0.04) after the first vaccine dose was statistically associated with a more sustained response and more gentle decline in antibody levels over time. No reported adverse events at the time of second vaccination were associated with differences in antibiotic dynamics (Figure 7).

## 4. Discussion

Healthcare workers are at the forefront of the COVID-19 response and may be at elevated risk of illness due to occupational exposure to SARS-CoV-2, in addition to risks conferred by more typical community-based transmission. The impact of COVID-19 illness in the healthcare workforce can present significant challenges in staffing medical facilities during times of increased concomitant community SARS-CoV-2 activity with associated increased healthcare utilization and hospitalizations. This cohort study of HCWs demonstrated significant short–term neutralizing antibody response in healthcare workers receiving the bnt162b2 vaccine and the mRNA-1273 vaccine during a period of high endemicity of COVID-19 using a semi-quantitative SARS-CoV2 cPass Surrogate Virus Neutralizing Test. Whereas commercially available test kits measure total antibody measurements towards spike proteins and/or nucleocapsid proteins, which may be a poor marker for neutralizing bio activity individuals [16], this study evaluated neutralizing antibodies for SARS-CoV-2 spike proteins, a gold standard for assessing antibody response to vaccination.

Similar to other cohorts [23,24], anti-spike protein antibody levels were noted to be transitory and decreased over time. Waning of antibody responses to SARS-CoV-2 vaccination [25] poses a risk to healthcare workers and health care institutions [26], leading to the strategy of booster vaccinations to maintain adequate protective antibody titers in this population. Similar to other published cohorts, demonstrating higher geometric mean titers of antibody in response to mRNA-1273 compared to Bnt162b2 [27,28], we demonstrated that healthcare workers vaccinated with mRNA-1273 were more likely to maintain a more sustained neutralizing antibody response compared to those vaccinated with Bnt162b2. There were no differences in antibody response characteristics based on gender.

A history of prior COVID-19 illness was associated with differences in antibody response and symptomatology after a primary vaccination series with both mRNA-1273 and bnt162b2. SARS-CoV-2 infection itself may lead to a more potent and durable immunologic stimulus then vaccination [29]. Our data are consistent with others [30,31,32,33,34,35] in that those with prior reported history of COVID-19 disease mounted higher antibody titers and progressed with a less steep decline of antibody titers over time after a primary vaccination series with either mRNA vaccine. Similar to other reports [36], women experienced a greater likelihood of adverse events and more severe adverse events after vaccinations than men.

The development of certain vaccine associated adverse events predicted a more durable antibody response to vaccination. It has previously been demonstrated that fever after vaccination may be associated with higher levels of COVID IgG [37] and other symptomatology after anti SARS-CoV-2 vaccination [38]. Furthermore, adverse events were most likely to be noted after the second exposure to the virus and/or viral vaccine components, with higher rates of adverse events noted in the first vaccination in those reporting prior clinical COVID-19 illness [34], as well as with the second vaccination in those reporting no such exposure.

Our research is subject to several limitations. The study was limited to medical workers at a single site. Males and the non-white population were under-represented in this study, and risk groups, such as the elderly over 70 years old and those with multiple comorbidities, were not included in the study. The number of subjects with a history of COVID-19 infection at baseline, or who had clinical breakthrough, was relatively low. Baseline antibody levels were not obtained. The evaluation was confined to data points obtained prior to widespread booster doses of vaccination for HCWs, which typically became available between Month 9 and Month 12 of follow-up for this HCW cohort. Other aspects of immune function, including NK cells [39] and spike specific T-cell immunity [40], were not evaluated, but they likely also play an important role in immunity to SARS-CoV-2.

## 5. Conclusions

These findings support the assertion that anti-spike mRNA vaccination leads to transitory but waning neutralizing antibody titers over time. Anti-spike vaccination with mRNA-1273 led to higher and more sustained neutralizing antibody responses compared to bnt162b2. The presence of certain vaccine-associated adverse events, either associated with second vaccination or with first vaccination in those with or without prior COVID infection, leads to a more blunted antibody decay curve and prolonged neutralized antibody response. Prior infection before first vaccination resulted in slower decay in antibody levels. This information may guide clinicians in discussing efficacy of mRNA vaccination strategies, especially in healthcare worker cohorts.

## Figures and Tables

**Table 1:** Baseline Characteristics of Study Participants.

	Breakthrough Infection (n = 8)		Non-Breakthrough Infection (n = 288)		All (n = 296)
	Baseline PCR Negative (n = 5)	Baseline PCR Positive (n = 3)	Baseline PCR Negative (n = 245)	Baseline PCR Positive (n = 43)	
Female	2 (40%)	1 (33%)	201 (82%)	33 (75%)	237 (80%)
Age (years)	38.4 ± 10.7	35.3 ± 6.1	44.2 ± 12.3	41.9 ± 11.9	43.7 ± 12.2
Ethnicity					
African American			5 (2%)		5 (2%)
Asian			16 (7%)	1 (2%)	17 (6%)
Caucasian	5 (100%)	3 (100%)	203 (83%)	37 (86%)	248 (83%)
Hispanic			15 (6%)	5 (11%)	20 (7%)
Vaccine Product					
mRNA-1273	3 (60%)	3 (100%)	105 (43%)	26 (60%)	137 (46%)
bnt162b2	2 (40%)		140 (57%)	17 (40%)	159 (54%)
Heart Disease			4 (2%)	1 (2%)	5 (2%)
Lung Disease			2 (1%)		2 (1%)
Diabetes			5 (2%)		5 (2%)
Obesity			9 (4%)		9 (3%)
Lifetime Smoking or Vaping history			9 (4%)	2 (5%)	11 (4%)
Pregnancy			1 (0%)		1 (0%)
Moderate or Severe Immunocompromise			8 (3%)		8 (3%)

**Table 2:** Slope Decline Categories Amongst Non-Breakthrough Infection Participants.

N (%)	Subjects without Prior COVID-19 History	Subjects with Prior COVID-19 History	Total
Slope with Gentle Decline	108 (44%)	39 (85%)	147 (50%)
Slope with Steep Decline	139 (56%)	7 (15%)	146 (50%)
Grand Total	247	46	293

**Table 3:** Slope Decline Categories in Subjects Reporting without Clinical COVID Illness prior to Vaccination.

N (%)	mRNA-1273	Bnt162b2	Total
Slope with Gentle Decline	64 (60%)	44 (31%)	108 (44%)
Slope with Steep Decline	43 (40%)	96 (69%)	139 (56%)
Total (%)	107	140	247

**Table 4:** Slope Decline Categories in Subjects Reporting Clinical COVID Illness prior to Vaccination.

N (%)	mRNA-1273	Bnt162b2	Total
Gentle Decline	24 (83%)	15 (88%)	39 (85%)
Steep Decline	5 (17%)	2 (12%)	7 (15%)
Total (%)	29	17	46

## Data Availability

The data presented in this study are available upon request from the corresponding author.
